# Structural insight into the recognition of *S*-adenosyl-L-homocysteine and sinefungin in SARS-CoV-2 Nsp16/Nsp10 RNA cap 2′-O-Methyltransferase

**DOI:** 10.1016/j.csbj.2020.09.032

**Published:** 2020-10-01

**Authors:** Panupong Mahalapbutr, Napat Kongtaworn, Thanyada Rungrotmongkol

**Affiliations:** a Department of Biochemistry, Faculty of Medicine, Khon Kaen University, Khon Kaen 40002, Thailand; b Program in Bioinformatics and Computational Biology, Graduate School, Chulalongkorn University, Bangkok 10330, Thailand; c Biocatalyst and Environmental Biotechnology Research Unit, Department of Biochemistry, Faculty of Science, Chulalongkorn University, Bangkok 10330, Thailand

**Keywords:** COVID-19, SARS-CoV-2, Nsp16/nsp10, Nucleoside analog, MD simulations, Rational drug design

## Abstract

The recent ongoing coronavirus disease 2019 (COVID-19) pandemic caused by severe acute respiratory syndrome coronavirus 2 (SARS-CoV-2) continues to rapidly spread across the world. To date, neither a specific antiviral drug nor a clinically effective vaccine is available. Among the 15 viral non-structural proteins (nsps), nsp16 methyltransferase has been considered as a potential target due to its crucial role in RNA cap 2′-O-methylation process, preventing the virus detection by cell innate immunity mechanisms. In the present study, molecular recognition between the two natural nucleoside analogs (*S*-adenosyl-l-homocysteine (SAH) and sinefungin (SFG)) and the SARS-CoV-2 nsp16/nsp10/^m7^G_ppp_AC_5_ was studied using all-atom molecular dynamics simulations and free energy calculations based on MM/GBSA and WaterSwap approaches. The binding affinity and the number of hot-spot residues, atomic contacts, and H-bond formations of SFG/nsp16 complex were distinctly higher than those of SAH/nsp16 system, consistent with the lower water accessibility at the enzyme active site. Notably, only SFG could electrostatically interact with the 2′-OH and N3 of RNA’s adenosine moiety, mimicking the methyl transfer reaction of *S*-adenosyl-l-methionine substrate. The atomistic binding mechanism obtained from this work paves the way for further optimizations and designs of more specific SARS-CoV-2 nsp16 inhibitors in the fight against COVID-19.

## Highlights

• The binding affinity towards SARS-CoV-2 nsp16 of SFG is higher than that of SAH.
• Asp99 is a key binding residue for SAH and SFG via charge-charge attraction.
• SFG could electrostatically interact with the 2′-OH and N3 groups of adenosine moiety of RNA substrate.
• The distance between 2′-OH of RNA and –NH_3_^+^ (at 6′ position) of SFG mimics the methyl transfer reaction.

## Graphical abstract

**Figure:**
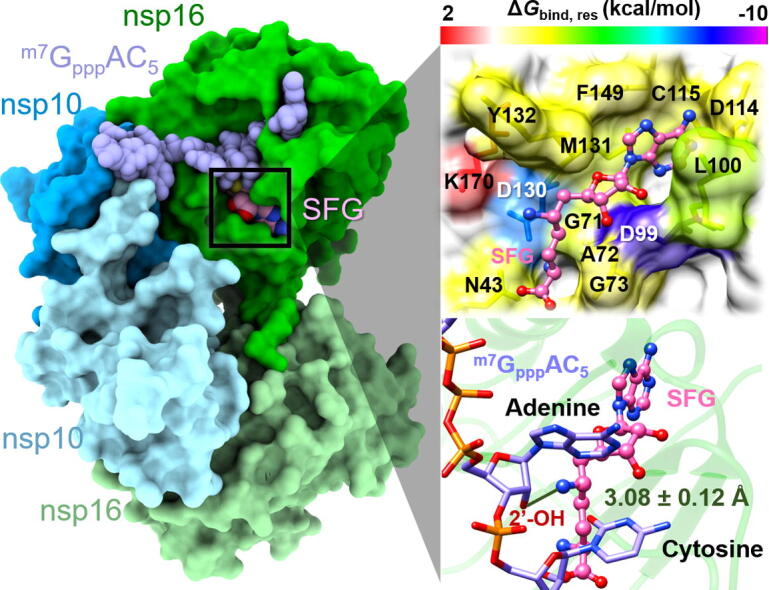

## 1 Introduction

The newly emerged coronavirus disease 2019 (COVID-19, designated by the World Health Organization (WHO) on February 11, 2020 [1]) caused by severe acute respiratory syndrome coronavirus 2 (SARS-CoV-2) was first reported in Wuhan city, Hubei province, China [2], [3]. This outbreak is epidemiologically associated with the Hua Nan seafood wholesale market; however, the exact origins of the infection are currently being investigated [4]. On 11 March 2020, WHO declared COVID-19 a global pandemic due to its rapid spread to more than 212 countries [5]. As of 22 May 2020, 4,995,996 confirmed cases and 327,821 deaths of COVID-19 were reported globally among 216 countries, and the number of new cases is rapidly increasing worldwide [6]. To date, neither a specific antiviral drug nor a clinically effective vaccine is available for the treatment and prevention of COVID-19 infections.

SARS-CoV-2, the seventh member of the family *Coronaviridae*, is an enveloped positive-strand RNA virus with a single-stranded genome of approximately 30 kb [7]. The life cycle of SARS-CoV-2 begins with the binding of its spike protein to the host cell receptor, namely angiotensin-converting enzyme 2 (ACE2) [8]. After viral entry through the endo-lysosomal pathway [9], the 5′-proximal two-thirds of the genome (open reading frames 1a and 1b) encodes two large overlapping replicase polyproteins (pp1a and pp1ab), which are further processed by viral proteases (i.e., 3-chymotrypsin-like protease (3CL_pro_) and papain-like protease (PL_pro_) [10]) to generate 15 non-structural proteins (nsps), termed nsp1 to nsp10 and nsp12 to nsp16 [4]. These cleavage products assemble into replicase-transcriptase complex (RTC) or function as accessory proteins in the viral replication process [11], [12].

**Fig. 1:**
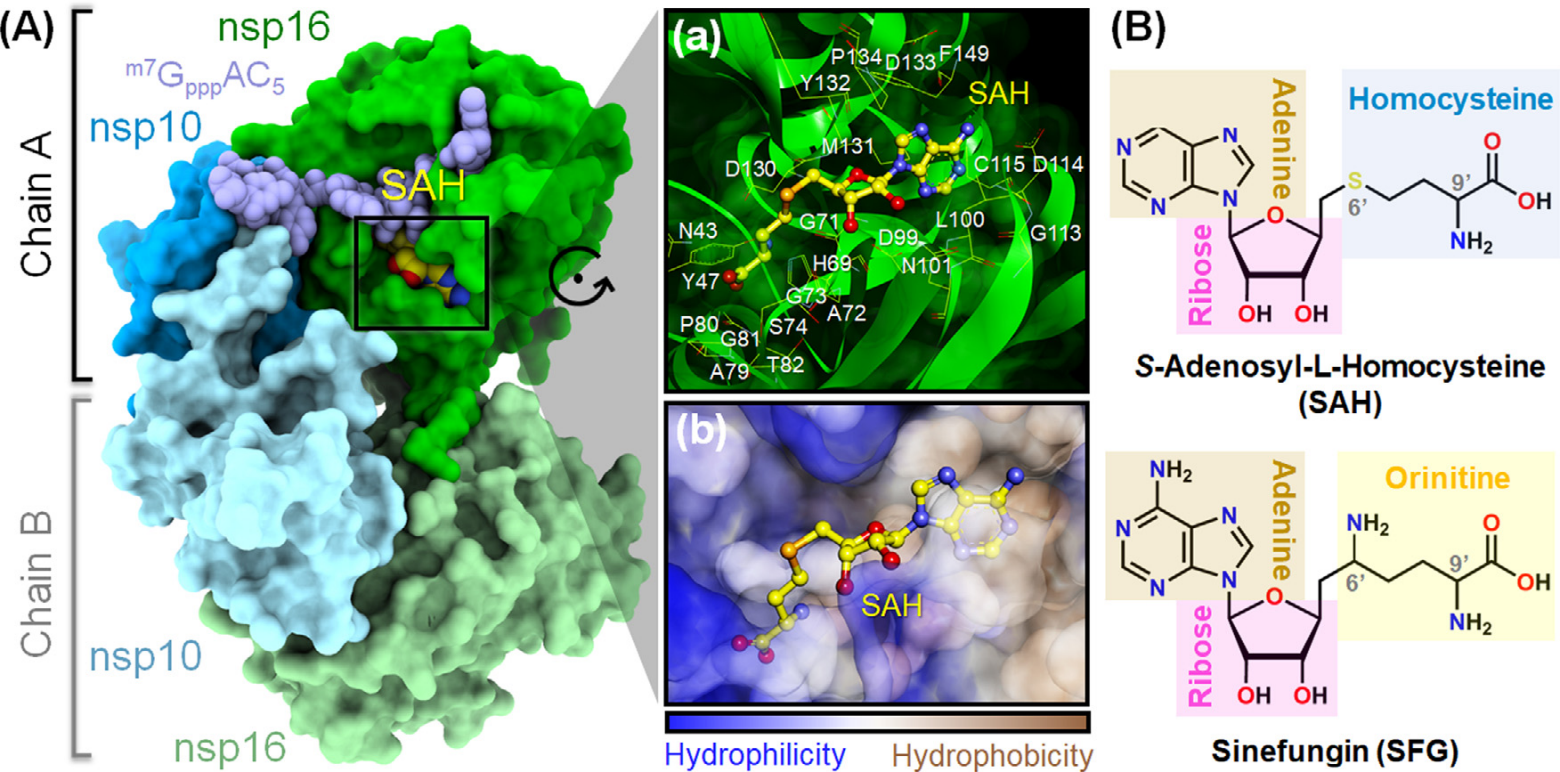
Three-dimensional structure of the SARS-CoV-2 nsp16/nsp10 heterodimer in complex with nucleoside analog SAH (PDB entry 6WQ3 [23]). The ^m7^G_ppp_AC_5_ RNA substrate is shown in pale purple sphere model. A close-up view of (a) amino acid residues and (b) hydrophobic (brown) and hydrophilic (blue) surfaces within 5 Å of SAH (yellow ball and stick model) in the 2′-O-MTase active site is illustrated in the right panel. (B) Two-dimensional chemical structures of the two studied nucleoside analogs SAH and SFG. (For interpretation of the references to colour in this figure legend, the reader is referred to the web version of this article.)

The RTC possesses catalytic activities required for viral genome synthesis and comprises most of the enzymes involved in 5′-guanosine cap formation [13], enhancing the mRNA stability and protecting mRNA from degradation by cellular 5′-3′ exoribonucleases [14]. According to CoV capping pathway [14], [15], the 5′-methylated-blocked cap structure is cotranscriptionally formed by four sequential enzymes: (i) an RNA triphosphatase (RTPase, nsp13) hydrolyzes the γ-phosphate of the nascent mRNA transcript, (ii) a guanylyltransferase (GTase) transfers a guanosine monophosphate (GMP) molecule to the 5′-diphosphate mRNA, forming a primitive cap structure (G_ppp_N…), (iii) a *S*-adenosyl-l-methionine (SAM)-dependent (N7-guanine)-methyltransferase (N7-MTase, nsp14) methylates guanine at the N7 position, producing a cap-0 structure (^m7^G_ppp_N…), and (iv) a SAM-dependent (nucleoside-2′-O-)-methyltransferase (2′-O-MTase, nsp16) together with its allosteric activator nsp10 [16] (Fig. 1A) further methylates the first transcribed nucleotide at the ribose 2′-OH position to form a cap-1 structure (^m7^G_ppp_N_2′-O-m_…). As the 2′-O-methylation process prevents (i) the detection of viral RNA by Mda5/RIG-I sensors and (ii) the inhibition of viral translation by the interferon-stimulated IFIT-1 protein [13], [17], [18], the nsp16 has been considered as a potential antiviral drug target [15], [19], [20].

Previous studies revealed that *S*-adenosyl-l-homocysteine (SAH, Fig. 1B), a by-product of methylation reaction, efficiently inhibits vaccinia virus (VV, *K_i_* = 0.53 μM [21]) and SARS-CoV (IC_50_ = 12 μM [16]) nsp16 MTase activities. In addition, sinefungin (SFG, Fig. 1B), a natural nucleoside analog of SAM, was found to be a potent nsp16 inhibitor against VV (*K_i_* = 75.2 nM [22]) and Middle East respiratory syndrome CoV (MERS-CoV, IC_50_ = 7.4 μM [13]). Interestingly, the nsp16 MTase inhibitory activity against SARS-CoV of SFG (IC_50_ = 736 nM) is ~16-fold greater than that of the SAH [16]. However, the inhibition of these two nucleoside analogs towards SARS-CoV-2 nsp16/nsp10 MTase has not yet been studied. In this work, several molecular modeling approaches were employed to investigate the structural dynamics and susceptibility of SAH and SFG against SARS-CoV-2 nsp16/nsp10 heterodimer based on the recently released crystal structure (PDB entry 6WQ3 [23]). It is our hope that the obtained structural and energetic information could be useful for rational drug design or development of novel nsp16 MTase inhibitors with higher binding efficiency to combat the COVID-19 pandemic.

## 2 Computational details

### 2.1 System preparation

The crystal structure of SARS-CoV-2 nsp16/nsp10 in complex with ^m7^G_ppp_A and SAH (PDB entry 6WQ3 [23]) as well as the atomic coordinate of SFG (PDB entry 6WKQ [24]) was retrieved from the RSCB Protein Data Bank. It should be noted that the research papers of these two crystal structures have not yet been published. To generate the ^m7^G_ppp_AC_5_ RNA hexamer model, the crystal structure of SARS-CoV-2 nsp16/^m7^G_ppp_A complex was aligned with that of the VV MTase VP39/^m7^G_ppp_A_6_ complex (PDB entry 1AV6 [25]). Then, the ^m7^G_ppp_A of SARS-CoV-2 nsp16 was linked to the A_5_ pentamer from the VV MTase at the 3′-OH position of A_1_ moiety using the Accelrys Discovery Studio 2.5^Accelrys Inc^ (DS2.5). After that, the generated model of ^m7^G_ppp_A_6_ in SARS-CoV-2 nsp16 was modified to ^m7^G_ppp_AC_5_ (according to the experimental 2′-O-MTase activity assays [15], [16], [26]) using macromolecules tool in DS2.5, and then the Dreiding-like forcefield in DS2.5 was employed to optimize the geometry of the modified C_5_ pentamer. The protonation states of SAH and SFG were calculated at pH 7.0 using MarvinSketch implemented in ChemAxon software [27], [28]. The PROPKA 3.0 web server [29] was used to assign the protonation states of all ionizable amino acids at pH 7.0, except for (i) the catalytic residue K46 that was set as the neutral form (LYN type of AMBER format) in accordance with the methyl transfer reaction mechanism [15] and (ii) the cysteine residues 74, 77, 90, 117, 120, 128, and 130 in the nsp10 monomers that were set as the deprotonated form (CYM type of AMBER format) for coordinating the Zn^2+^ ions [12]. To prepare the partial atomic charges and parameters of ligands, all of the structures were fully optimized by means of the HF/6-31G* level of theory using Gaussian09 program [30] as previously described [31], [32], [33]. The electrostatic potential (ESP) charges were subsequently computed with the same method and basis set. The antechamber package was used to convert ESP charges to restrained ESP (RESP) charges. The AMBER OL3 force field [34] and the general AMBER force field version 2 (GAFF2) [35] were adopted for ^m7^G_ppp_AC_5_ parameters. Missing hydrogen atoms were added using the LEaP module implemented in AMBER16. The AMBER ff14SB force field was applied for the protein [36]. Subsequently, each system was solvated using the TIP3P water model [37] with a spacing distance of 10 Å between the solvation box edge and the protein surface, and the total charge of system was neutralized by incorporating sodium ions. The added hydrogen atoms and water molecules were then minimized using 1000 steps of steepest descent followed by 2500 steps of conjugated gradient methods. Finally, the whole system was minimized using the same minimization procedure.

### 2.2 Molecular dynamics (MD) simulations and structural analyses

Each simulated system was performed under the periodic boundary condition with the isothermal–isobaric (*NPT*) ensemble. The particle mesh Ewald summation method [38] was employed to treat the charge-charge interactions, while a cutoff of 10 Å was set for non-bonded interactions. The SHAKE algorithm [39] was used to constrain all covalent bonds involving hydrogen atoms. In the relaxation phase, all of the models were gradually heated up from 10 to 310 K for 100 ps with an application of a harmonic restraint of 30.0 kcal/mol·Å^2^ to the protein–ligand complex. In the next equilibrium phase, each complex was subjected to restrained MD simulations at 310 K with the harmonic restraint of 30, 20, 10, 5, and 2.5 kcal/mol·Å^2^ for 500 ps in total followed by unrestrained MD at 310 K for 500 ps. Subsequently, MD simulations with a time step of 2 fs were performed under the *NPT* ensemble (310 K and 1 atm) until reaching 50 ns. MD simulation of each complex was performed in triplicate (MD1-3). The CPPTRAJ module [40] of AMBER16 was used to compute the structural information. The hydrogen bond (H-bond) formation was calculated using the two structural criteria: (i) distance between H-bond donor (HBD) and acceptor (HBA) ≤ 3.5 Å and (ii) the angle of HBD–H⋯HBA ≥ 120°.

### 2.3 Free energy calculations

The binding free energy (Δ*G*_bind_) and per-residue decomposition free energy ($\Delta G_{\text{bind}}^{\text{residue}}$) were calculated using the molecular mechanics/generalized Born surface area (MM/GBSA) method [41], [42] on 100 MD snapshots extracted from the last 20 ns of the MD production phase. Note that only nsp16/ligand/RNA ternary complex in chain A was considered for free energy calculations.

The Δ*G*_bind_ consists of the molecular mechanics energy (Δ*E*_MM_) in gas phase, solvation free energy (Δ*G*_solv_), and entropy term (Δ*S*) as given in Eq. (1).

(1) $$\Delta G_{\text{bind}}=\Delta E_{\text{MM}}+\Delta G_{\text{solv}}-T\Delta S$$

The Δ*E*_MM_ was obtained by combining electrostatic (Δ*E*_ele_) and van der Waal (Δ*E*_vdW_) energies between ligand and its receptor, whereas the Δ*G*_solv_ was calculated according to Eq. (2).

(2) $$\Delta G_{\text{solv}}=\Delta G_{\text{solv}}^{\text{ele}}+\Delta G_{\text{solv}}^{\text{nonpolar}}$$

The ${\Delta G}_{\text{solv}}^{\text{ele}}$ was estimated using the GB equation [41], while the ${\Delta G}_{\text{solv}}^{\text{nonpolar}}$ was derived from SASA calculation [43] as shown in Eq. (3), where *γ* and *b* are the experimental solvation parameters equal to 0.00542 kcal/mol·Å^2^ and 0.92 kcal/mol, respectively [44].

(3) $${\Delta G}_{\text{solv}}^{\text{nonpolar}}=\gamma SASA+b$$

In addition to MM/GBSA technique, the WaterSwap method [45], [46] was employed to calculate the Δ*G*_bind_ by swapping the ligand bound to the protein with an equivalent volume of explicit water molecules in the protein-binding pocket using a replica-exchange thermodynamic integration algorithm. The final MD snapshot of each system was used for WaterSwap calculations (1000 iterations) without considering the RNA molecule. The absolute binding affinity of each model was calculated by averaging Δ*G*_bind_ values obtained from four different statistical techniques, including thermodynamic integration (TI), Bennett’s acceptance ratio, free energy perturbation, and quadrature-based integration of TI.

## 3 Results and discussion

### 3.1 System stability

**Fig. 2:**
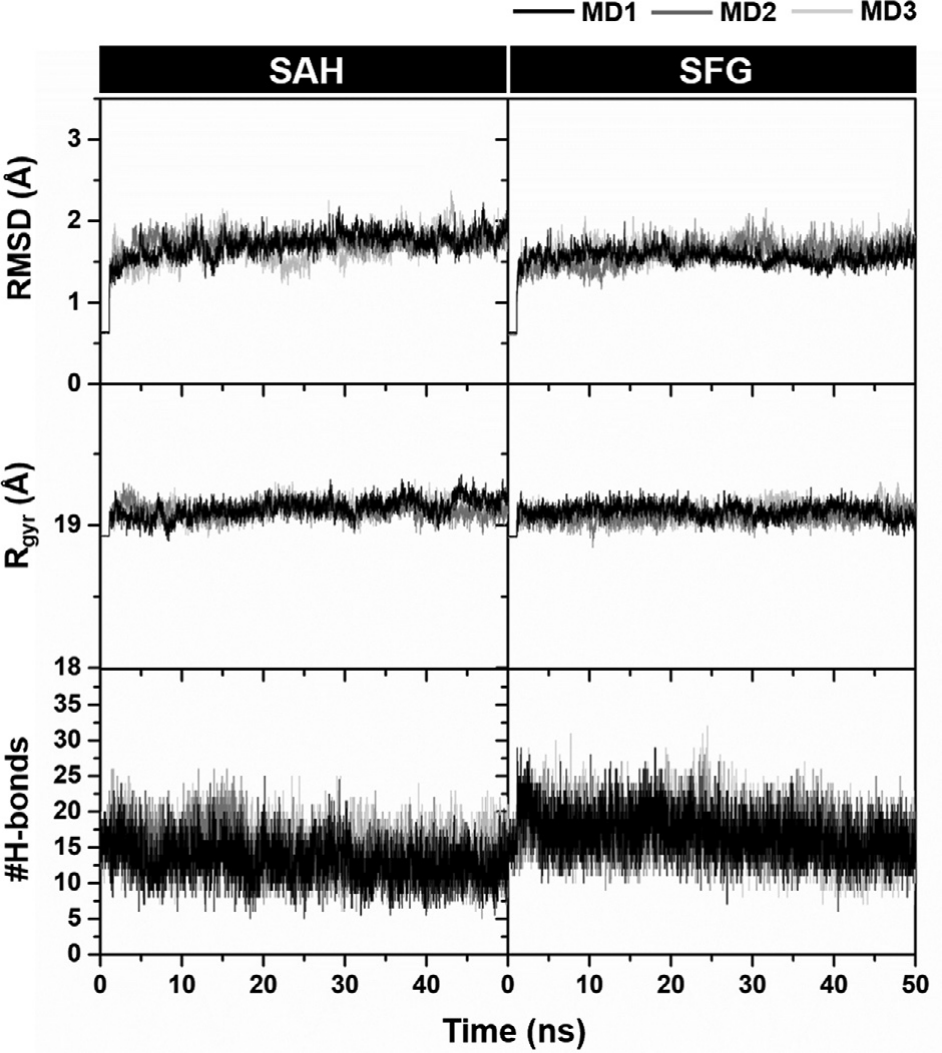
Time evolution of (top) RMSD of residues around 5 Å of the ligand, (middle) R_gyr_ of nsp16 in chain A, and (bottom) #H-bonds of SAH (left) and SFG (right) in complex with SARS-CoV-2 nsp16/nsp10/^m7^G_ppp_AC_5_ for three independent simulations (MD1-3).

The stability of each simulated model was determined using the calculations of root mean square displacement (RMSD), radius of gyration (R_gyr_), and the number of intermolecular H-bonds (#H-bonds) along the simulation time. As shown in Fig. 2, the RMSD and R_gyr_ values of both SAH and SFG systems dramatically increased in the first 5 ns and then maintained at a fluctuation of ~1.5–2.0 Å and ~19.0 Å, respectively until the end of simulation time for all independent runs. In the case of time evolution of #H-bonds, we found the moderate fluctuation during the first 25 ns and then all of the systems reached the equilibrium state after 25 ns. Notably, the #H-bonds of SFG system (15.75 ± 0.98 over the last 20 ns) was higher than that of the SAH model (13.27 ± 1.17), suggesting that SFG was more susceptible to the SARS-CoV-2 nsp16/nsp10 (discussed in more detail later). In this work, the MD trajectories from 30 to 50 ns were extracted for further analysis in terms of: (i) the binding affinity between SAH/SFG and SARS-CoV-2 nsp16, (ii) hot-spot residues involved in ligand binding, (iii) protein–ligand H-bonding, and (iv) solvent accessibility and atomic contact at the enzyme active site.

### 3.2 Predicted inhibitory efficiency

The susceptibility of SAH and SFG to SARS-CoV-2 2′-O-MTase was estimated using Δ*G*_bind_ calculations based on MM/GBSA and WaterSwap methods. As shown in Table 1, the Δ*E*_MM_ estimations in gas phase revealed that electrostatic attraction is the main force inducing molecular complexation with the SARS-CoV-2 nsp16/nsp10/^m7^G_ppp_AC_5_ of both SAH (Δ*E*_ele_ of −116.06 ± 8.87 kcal/mol) and SFG (−508.88 ± 6.58 kcal/mol) and is ~2–10-fold stronger than the van der Waals (vdW) interaction (Δ*E*_vdW_ of −51.17 ± 3.04 and −49.67 ± 2.85 kcal/mol for SAH and SFG, respectively). This finding is in good agreement with the reported binding of SAH and SFG to the flavivirus MTase [47] as well as with the predicted interactions of SAM/SARS-CoV-2 nsp16 complex by the adaptive Poisson-Boltzmann solver program [12] and the electrostatic potential surface calculations at the active site of SARS-CoV 2′-O-MTase in complex with SAM [48]. Notably, the electrostatic contribution of SFG system was ~4-fold higher than that of SAH model, since the positively charged –NH_3_^+^ group at 6′ position of SFG could electrostatically interact with the adenosine moiety of RNA substrate (discussed in more details later).

**Table 1:** Average Δ*G*_bind_ and its energy components (kcal/mol) of SAH and SFG in complex with SARS-CoV-2 nsp16/nsp10/^m7^G_ppp_AC_5_ calculated with the MM/GBSA and WaterSwap methods. Data are shown as mean ± standard deviation (SD) of three independent simulations.

Energy component	SAH	SFG
**MM/GBSA**		
Δ*E*_vdW_	−51.17 ± 3.04	−49.67 ± 2.85
Δ*E*_ele_	−116.06 ± 8.87	−508.88 ± 6.58
Δ*E*_MM_	−167.23 ± 6.75	−558.55 ± 5.06
$\Delta G_{\mathrm{solv}\;}^{\mathrm{ele}}$	124.96 ± 7.44	497.62 ± 2.83
$\Delta G_{\mathrm{solv}}^{\mathrm{nonpolar}}$	−6.76 ± 0.07	−6.87 ± 0.28
−*T*Δ*S*	28.88 ± 0.79	30.60 ± 0.61
Δ*G*_bind_	−20.15 ± 3.16	−37.19 ± 4.86

**WaterSwap**		
Δ*G*_bind_	−18.31 ± 3.95	−28.05 ± 1.05

There were evidences that the inhibitory activity against VV, MERS-CoV, and SAR-CoV nsp16 MTases of SFG is higher than that of SAH [13], [16], [22]. In correlation with these reports, the Δ*G*_bind_ calculations showed that SFG (Δ*G*_bind_ of −37.19 ± 4.86 kcal/mol for MM/GBSA and −28.05 ± 1.05 kcal/mol for WaterSwap) has significantly greater binding affinity than SAH (−20.15 ± 3.16 kcal/mol for MM/GBSA and −18.31 ± 3.95 kcal/mol for WaterSwap) towards SARS-CoV-2 nsp16/nsp10. Thus, based on these evidences, SFG is suggested to use as a SARS-CoV-2 nsp16 inhibitor to combat the COVID-19.

### 3.3 Hot-spot residues

**Fig. 3:**
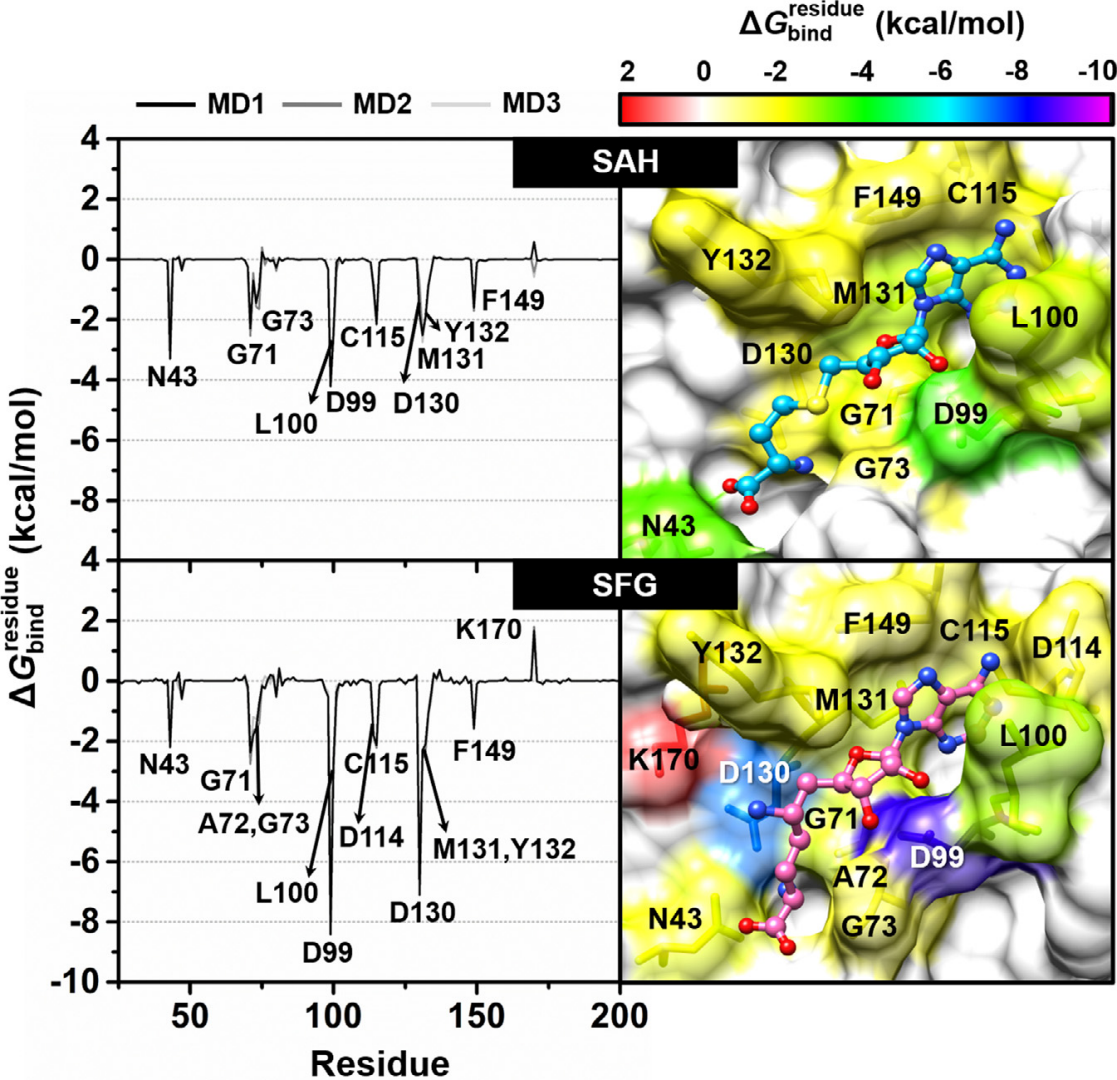
(Left) $\Delta G_{\text{bind}}^{\text{residue}}$ of SAH (top) and SFG (bottom) in complex with SARS-CoV-2 nsp16/nsp10. (Right) Representative 3D structures showing the ligand orientation in the enzyme active site drawn from MD1. The contributing residues involved in the binding of the two studied nucleoside analogs are colored according to their $\Delta G_{\text{bind}}^{\text{residue}}$ values, where the highest to lowest free energies are shaded from red to magenta, respectively. (For interpretation of the references to colour in this figure legend, the reader is referred to the web version of this article.)

To investigate crucial amino acid residues associated with the binding of the two studied nucleoside analogs at the active site of SARS-CoV-2 nsp16, the $\Delta G_{\text{bind}}^{\text{residue}}$ calculation based on MM/GBSA method was conducted. The total energy contribution from each residue involved in ligand binding of both systems is plotted in Fig. 3, in which the negative and positive $\Delta G_{\text{bind}}^{\text{residue}}$ values denote energy stabilization and destabilization, respectively. It should be note that (i) among residues 1 to 298 of nsp16, only residues 25 to 200 were illustrated, (ii) residues exhibiting the energy stabilization of ≤−1.5 kcal/mol and energy destabilization of >1 kcal/mol were herein taken into account, and (iii) only data derived from MD1 were discussed below for simplification.

**Fig. 4:**
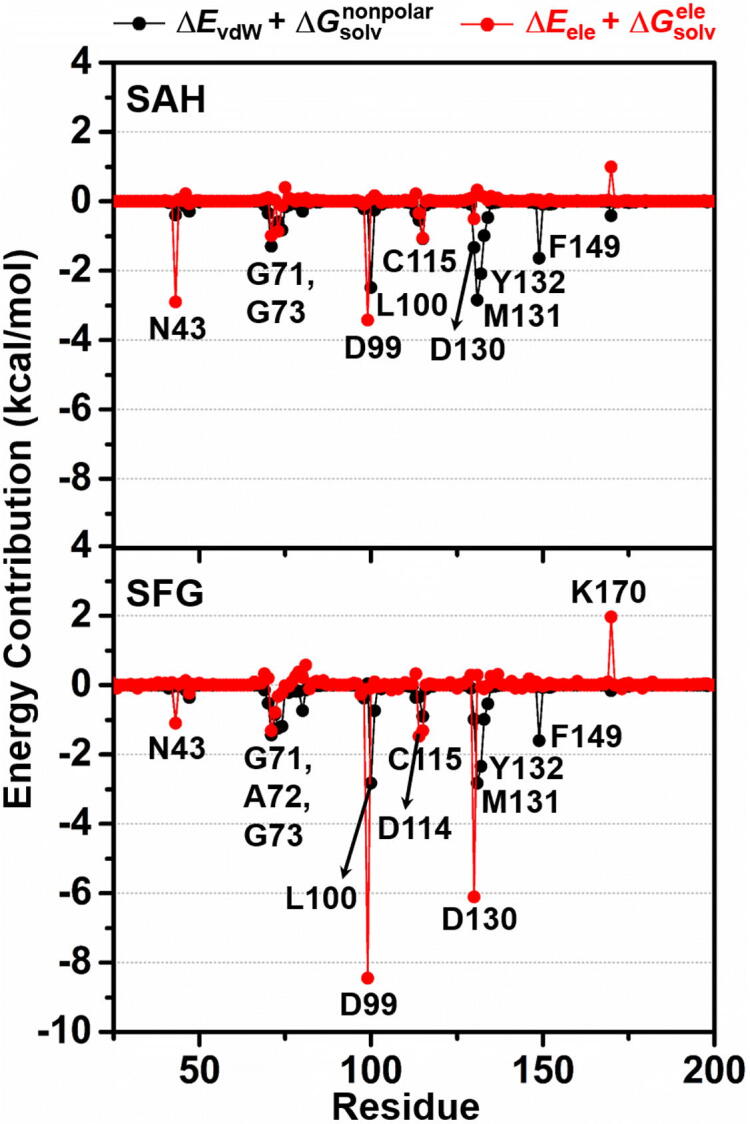
Electrostatic (Δ*E*_ele_ + ${\Delta G}_{\text{solv}}^{\text{ele}}$, red) and vdW (Δ*E*_vdW_ + ${\Delta G}_{\text{solv}}^{\text{nonpolar}}$, black) energy contributions from each residue of SARS-CoV-2 nsp16 to the binding of SAH (top) and SFG (bottom) derived from MD1. (For interpretation of the references to colour in this figure legend, the reader is referred to the web version of this article.)

**Fig. 5:**
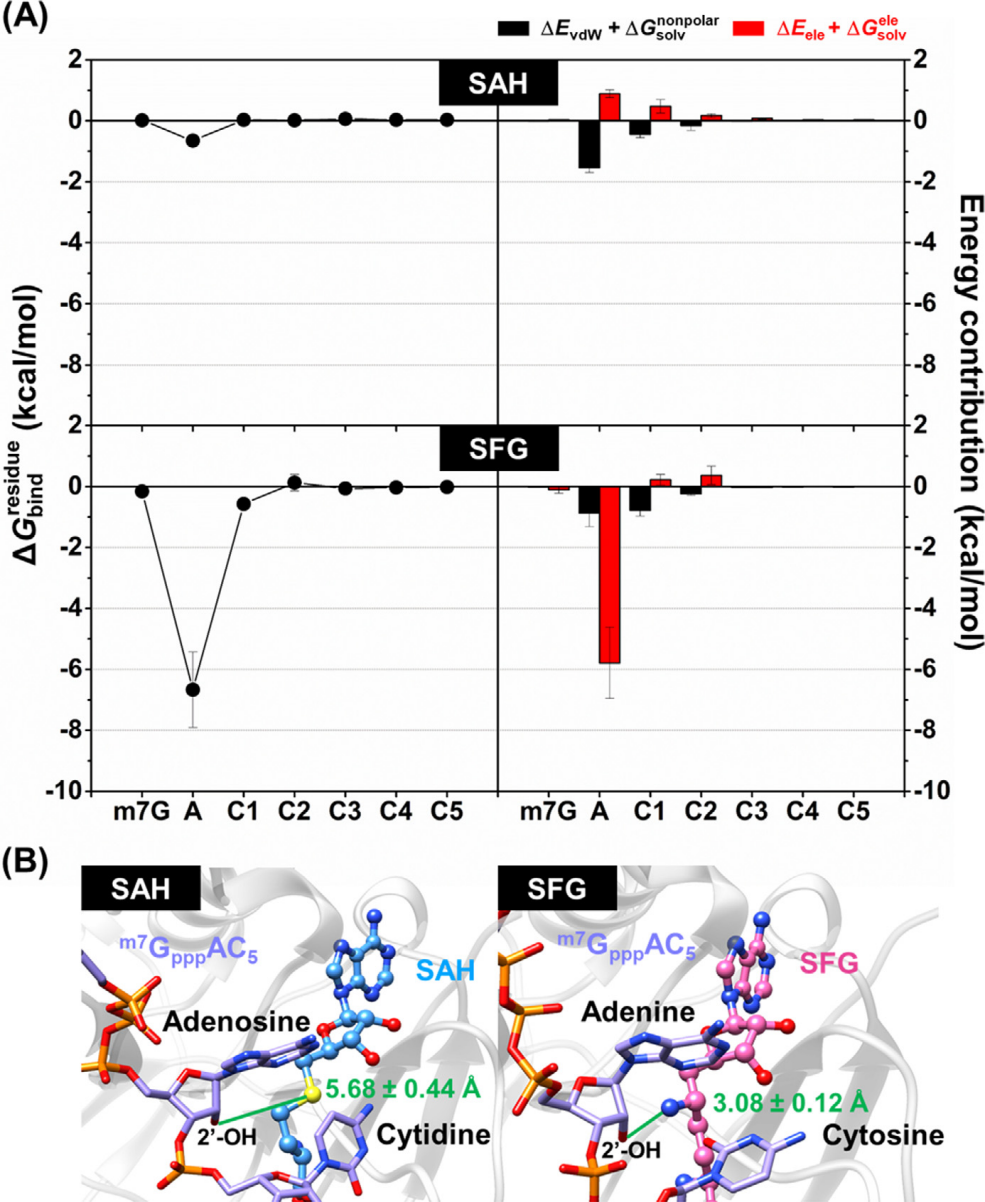
(A) $\Delta G_{\text{bind}}^{\text{residue}}$ (left) as well as electrostatic (Δ*E*_ele_ + ${\Delta G}_{\text{solv}}^{\text{ele}}$, red) and vdW (Δ*E*_vdW_ + ${\Delta G}_{\text{solv}}^{\text{nonpolar}}$, black) energy contribution from MD1 (right) of the ^m7^G_ppp_AC_5_ RNA substrate towards the binding of SAH (top) and SFG (bottom) to the SARS-CoV-2 nsp16/nsp10. Data are shown as mean ± SD of three independent simulations. (B) Representative 3D structures showing the distance (green line) between the sulfur atom of SAH (left) or the nitrogen atom of SFG (right) at 6′ position and the 2′-oxygen atom of RNA’s adenosine moiety in the enzyme active site. Distances are shown as mean ± SD (n = 3) calculated from the final snapshot of each complex. (For interpretation of the references to colour in this figure legend, the reader is referred to the web version of this article.)

**Fig. 6:**
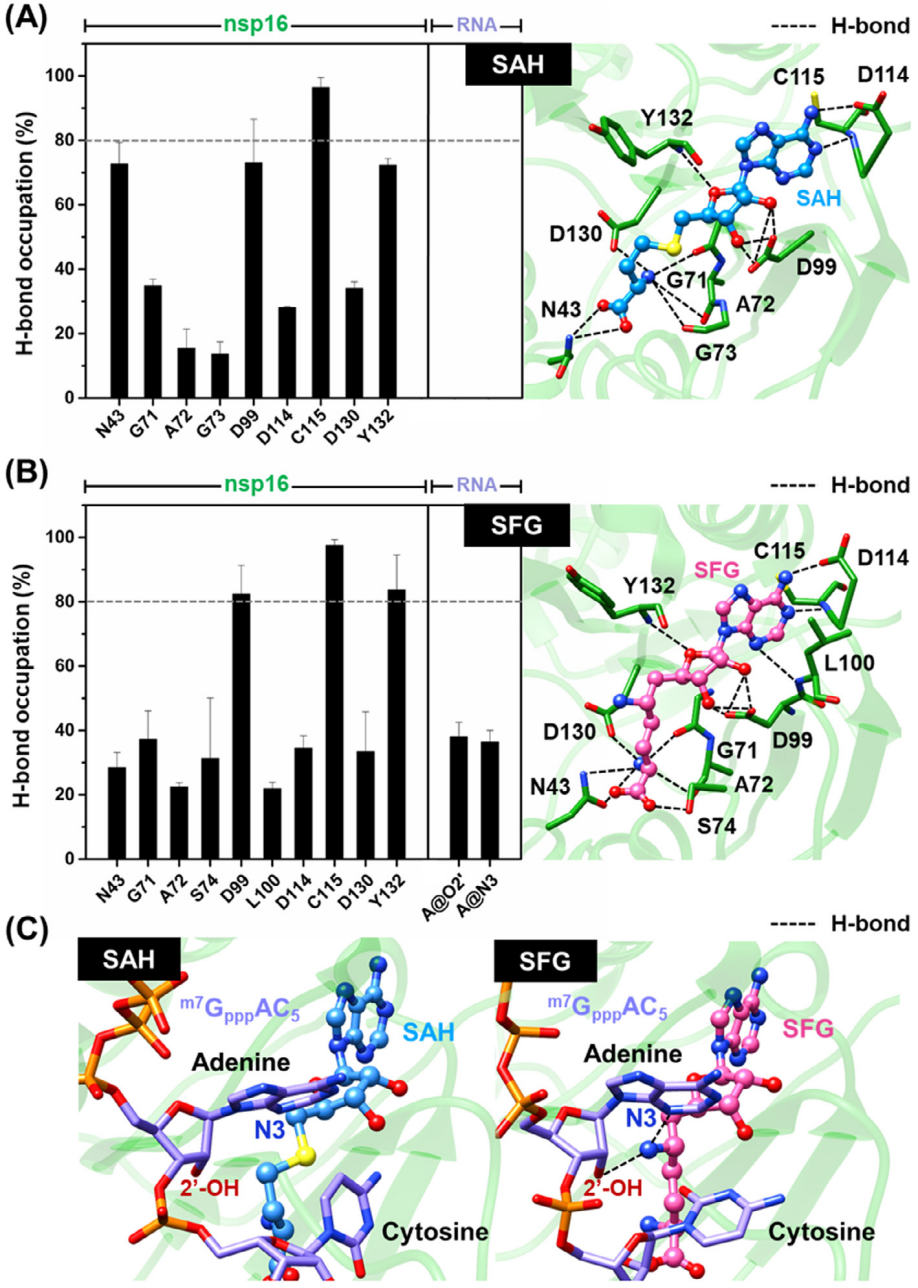
Percentage of H-bond occupation of SARS-CoV-2 nsp16 contributing to the binding of (A) SAH and (B) SFG, where the ligand orientation in the enzyme active site is illustrated in the right panel. (C) H-bond formation between (left) SAH or (right) SFG and the RNA substrate in the active site of SARS-CoV-2 nsp16. Black dashed line indicates H-bond formation. Data are shown as mean ± SD of three independent simulations.

The obtained results demonstrated that there were 10 (N43, G71, G73, D99, L100, C115, D130, M131, Y132, and F149) and 12 residues (N43, G71, A72, G73, D99, L100, D114, C115, D130, M131, Y132, and F149) associated with the binding of SAH and SFG, respectively. The higher contributing residues found in SFG/SARS-CoV-2 nsp16 complex was in good agreement with the time evolution of #H-bonds (Fig. 2) as well as the Δ*G*_bind_ (Table 1) results as mentioned earlier. Among the 10–12 hot-spot residues, D99 showed the lowest energy contribution for both SAH (−3.58 kcal/mol) and SFG (−8.41 kcal/mol) *via* electrostatic attraction (Fig. 4) and H-bond formation (Fig. 6, discussed later), suggesting the most important key binding residue. To support this finding, a model of D99A mutant SARS-CoV-2 nsp16 in complex with both ligands was constructed and then subjected to MD simulations and free energy calculations. The obtained results demonstrated that the D99A mutation dramatically decreased not only the susceptibility (by ~2 to 6 kcal/mol) but also the $\Delta G_{\text{bind}}^{\text{residue}}$ values from several hot-spot residues, including N43, D99A, Y132, and F149 to the binding of both nucleosides compared to the wild-type SARS-CoV-2 nsp16 (Fig. S1). However, further experimental validation (e.g., site-directed mutagenesis) should be conducted. In addition to D99, residues L100, C115, M131, and F149 were found to stabilize the adenine ring of SAH and SFG through vdW forces (up to ~ −3.0 kcal/mol, Fig. 4). These findings are in good agreement with the reported binding of SAM to the SARS-CoV and SARS-CoV-2 nsp16/nsp10 MTases showing that the 2′- and 3′–OH moieties of SAM’s adenosine ribose are stabilized by the D99 residue through H-bonds, whereas SAM's adenine ring is hydrophobically stacked by the L100, C115, M131, and F149 residues [12], [48]. Notably, both SAH and SFG could interact with the D130, one of the catalytic tetrad residues (K46, D130, K170, and E203 [15]), with the $\Delta G_{\text{bind}}^{\text{residue}}$ of −1.82 and −7.11 kcal/mol, respectively. However, the energy destabilization ($\Delta G_{\text{bind}}^{\text{residue}}$ of 1.67 kcal/mol) was detected between the catalytic K170 residue and the –NH_3_^+^ group at 6′ position of SFG due to the positive charge repulsion (Fig. 3 and Fig. 4). Therefore, we suggest to modify this positively charged amino group of SFG to other polar moieties (e.g., halogen atoms, carboxylate, etc.) in order to impair charge-charge repulsion. However, it is worth noting that the amino group at 6′ position of SFG strongly interacted with the 2′-O and N3 atoms of adenosine moiety of RNA substrate *via* electrostatic contributions and H-bond formations (Fig. 5 and Fig. 6); thus, chemical modifications of this part need to consider such crucial point. The importance of 6′ position for MTase inhibitory activity is evidently supported by the previous studies demonstrating that the introduction of functional group(s) at C6′ of SAM and SFG could potentially and selectively inhibit the nicotinamide *N*‑methyltransferase (NNMT) [49] and protein lysine methyltransferase SETD2 enzymes [50], respectively.

In terms of the contribution from the vdW (Δ*E*_vdW_ + ${\Delta G}_{\text{solv}}^{\text{nonpolar}}$, black line) and electrostatic (Δ*E*_ele_ + ${\Delta G}_{\text{solv}}^{\text{ele}}$, red line) energies from each residue, it can be clearly seen from Fig. 4 that the main energy contribution for stabilizing both nucleoside analogs was the electrostatic energy (up to ~ −8.0 kcal/mol), especially for the residues N43, D99, and D130. Whereas, the vdW contribution was observed in the range of ~0.0 to −3.0 kcal/mol as related to the Δ*E*_MM_ results (Table 1).

From the $\Delta G_{\text{bind}}^{\text{residue}}$ calculation between ^m7^G_ppp_AC_5_ RNA substrate and nucleoside analog(s) (Fig. 5A), we found that only adenosine moiety of RNA plays a major role in the recognition of both inhibitors. Notably, the $\Delta G_{\text{bind}}^{\text{residue}}$ of adenosine moiety in SFG system (−6.66 ± 1.23 kcal/mol) was ~10-fold lower than that in SAH model (−0.65 ± 0.03 kcal/mol). This is because the positively charged –NH_3_^+^ group at 6′ position of SFG (instead of the –S– moiety of SAH) could electrostatically interact with the adenosine moiety of the RNA substrate (Δ*E*_ele_ + ${\Delta G}_{\text{solv}}^{\text{ele}}$of ~ −6 kcal/mol, red). More importantly, the distance between the nitrogen atom of amino group at 6′ position of SFG and the adenosine ribose 2′-oxygen atom of RNA (3.08 ± 0.12 Å, Fig. 5B) approximates the donor–acceptor distance in methyl transfer reaction of the SAM substrate [15]. Altogether, these evidences suggested that the modification of –S– moiety of SAH to –NH_3_^+^ group dramatically increased the binding interactions towards SARS-CoV-2 nsp16, especially electrostatic forces at the adenosine moiety of the RNA substrate.

### 3.4 Protein-ligand hydrogen bonding

Since electrostatic interaction was the main force inducing protein–ligand complexations (Table 1), we further investigated the structural insights into the intermolecular H-bond formation between SAH/SFG and SARS-CoV-2 nsp16 during the last 20-ns simulations using the defining criteria described in material and method section. The average percentage of H-bond occupations calculated from the three independent simulations is illustrated in Fig. 6.

As expected, the two focused ligands formed H-bonds with several polar and charged residues in the enzyme active site. Notably, the number of (i) amino acid residues responsible for H-bond formations and (ii) strong H-bonds (>80% occupancy) in the SFG/nsp16 complex (10 residues: N43, G71, A72, S74, D99, L100, D114, C115, D130, and Y132 as well as three strong H-bonds at D99, C115, and Y132) was higher than that in the SAH/nsp16 system (9 residues: N43, G71, A72, G73, D99, D114, C115, D130, and Y132 as well as one strong H-bond at C115), suggesting that SFG interacted with the SARS-CoV-2 nsp16 better than SAH, in line with the Δ*G*_bind_ calculations (Table 1). It has been reported that the conserved residues N43, Y47, G71, A72, S74, G81, D99, N101, L100, D114, and M131 play an important role in coordinating the recognition of SAM substrate in the active site of SARS-CoV nsp16 *via* H-bonds [12]. Corresponding with this evidence, the aforementioned residues were also involved in H-bond formations between the SAH/SFG and the SARS-CoV-2 nsp16 as follows: (i) N43, G71, A72, G73, S74 and D130, (ii) D99, (iii) Y132, and (iv) L100, C115, and D114 residues formed H-bonds with the –COO^−^ and –NH_3_^+^ (at C9′ position) groups, the 2′- and 3′-OH moieties, the ether oxygen atom on ribose ring, and the nitrogen atoms on adenine ring of both ligands, respectively. Apart from protein-ligand H-bonding, the –NH_3_^+^ group at 6′ position of SFG could form H-bonds with the 2′-O and N3 atoms of adenosine moiety of ^m7^G_ppp_AC_5_ RNA substrate, mimicking the position of the methyl group of SAM during the methyl transfer reaction [15] (Fig. 5B).

### 3.5 Solvent accessibility and atomic contact at the enzyme active site

**Fig. 7:**
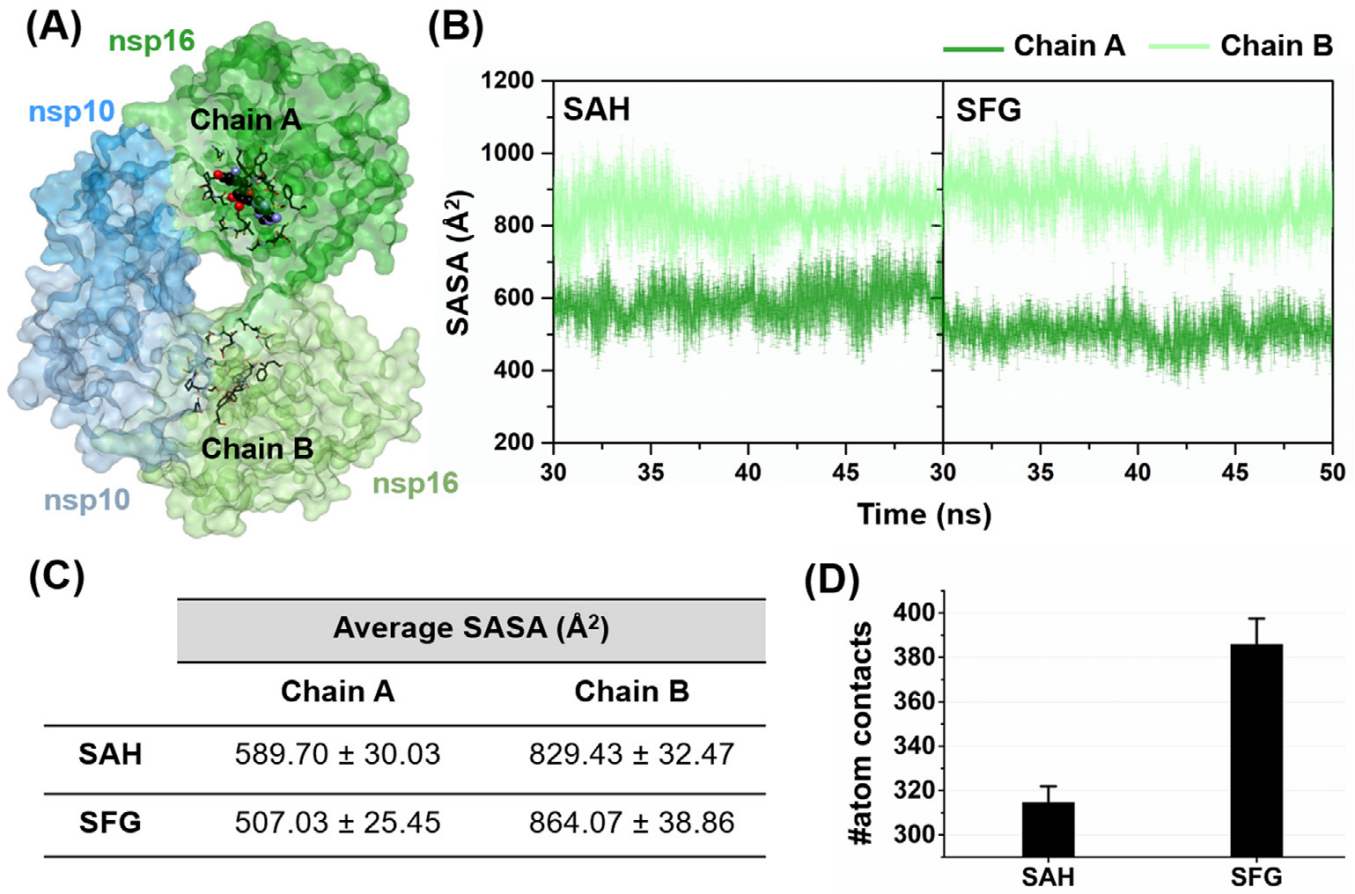
(A) SARS-CoV-2 nsp16/nsp10 heterodimer, in which chain A with a ligand bound and chain B without a ligand bound are shown in shades of dark green and pale green, respectively. Note that the amino acid residues within 5 Å (stick model) of the ligand (Corey-Pauling-Koltun (CPK) representation) were used for SASA calculations. (B) Time evolution of average SASA for the two studied systems. (C) Average SASA of SAH and SFG systems during the last 20 ns. (D) The number of atomic (native + non-native) contacts involved in the complexation between the nucleoside analog(s) and the SARS-CoV-2 nsp16 during the last 20-ns MD simulations. Data are shown as mean ± SD of three independent simulations. (For interpretation of the references to colour in this figure legend, the reader is referred to the web version of this article.)

To characterize the effect of water accessibility on the SARS-CoV-2 nsp16 active site, the solvent-accessible surface area (SASA) calculations were performed on the residues within 5 Å of each nucleoside inhibitor (Fig. 7A). Note that our complex model contained the ligand binding only to chain A. As shown in Fig. 7B–C, the SASAs for the apo protein (chain B, pale green) were 829.43 ± 32.47 and 864.07 ± 38.86 Å^2^ for SAH and SFG systems, respectively. Upon molecular complexation with the nucleoside analogs (chain A, dark green), the SASAs of both models tremendously decreased by ~200 to 350 Å^2^, consistent well with the reported SASA loss during the binding process from other works [51], [52], [53]. It is worth noting that the SFG model (507.03 ± 25.45 Å^2^) exhibited lower SASAs than the SAH system (589.70 ± 30.03 Å^2^), indicating that the binding efficiency of SFG is greater than that of SAH, as evidenced by the Δ*G*_bind_ calculations (Table 1).

We further characterized the number of atomic contacts (#atom contacts) within 5 Å of each ligand during the last 20-ns simulations. The average results from triplicate MD runs (Fig. 7D) demonstrated that the SFG system (385.95 ± 11.55) showed higher #atom contacts than the SAH model (314.62 ± 7.39). Taken together, the native contact results consistently support the SASA and Δ*G*_bind_ calculations.

## 4 Conclusion

In this work, the binding pattern and susceptibility of the two nucleoside analogs SAH and SFG against SARS-CoV-2 nsp16/nsp10/^m7^G_ppp_AC_5_ were fully revealed by all-atom MD simulations and free energy calculations based on MM/GBSA and WaterSwap methods. According to the Δ*G*_bind_ prediction, the susceptibility to the SARS-CoV-2 nsp16 of SFG was significantly higher than that of SAH, consistent with the lower water accessibility at the enzyme active site as well as with the higher number of H-bond formations, hot-spot residues, and atomic contacts. The D99 residue showed the lowest $\Delta G_{\text{bind}}^{\text{residue}}$ for the binding of both nucleoside inhibitors *via* electrostatic attractions and H-bond formations, whereas L100, C115, M131, and F149 residues stabilized the adenine ring of ligands through vdW forces. Notably, only SFG could electrostatically interact with the 2′-OH and N3 of RNA’s adenosine moiety, mimicking the methyl transfer reaction of SAM substrate. Altogether, the fundamental knowledge at the atomic level from this work could be helpful for further design and development of more specific SARS-CoV-2 nsp16 inhibitors in the fight against COVID-19.

## CRediT authorship contribution statement

**Panupong Mahalapbutr:** Conceptualization, Data curation, Formal analysis, Investigation, Methodology, Validation, Visualization, Writing - original draft, Writing - review & editing. **Napat Kongtaworn:** Formal analysis, Methodology, Validation. **Thanyada Rungrotmongkol:** Funding acquisition, Project administration, Resources, Software, Supervision, Visualization, Writing - review & editing.

## Declaration of Competing Interest

The authors declare that they have no known competing financial interests or personal relationships that could have appeared to influence the work reported in this paper.
