# Supplementary material for: Structural insight into the recognition of S-adenosyl-L-homocysteine and sinefungin in SARS-CoV-2 Nsp16/Nsp10 RNA cap 2′-O-Methyltransferase
Source: Comput Struct Biotechnol J. 2020 Oct 1;18:2757–65. doi: 10.1016/j.csbj.2020.09.032 (PMC7527316; doi:10.1016/j.csbj.2020.09.032)
Supplement: Supplementary data 1 [file mmc1.docx]

**Supplementary data**

**Structural Insight into the Recognition of *S*-Adenosyl-L-Homocysteine and Sinefungin in SARS-CoV-2 Nsp16/Nsp10 RNA Cap 2’-O-Methyltransferase**

Panupong Mahalapbutr^1^, Napat Kongtaworn^2^, and Thanyada Rungrotmongkol^2,3,^*

*^1^Department of Biochemistry, Faculty of Medicine, Khon Kaen University, Khon Kaen 40002, Thailand*

*^2^Program in Bioinformatics and Computational Biology, Graduate School, Chulalongkorn University, Bangkok 10330, Thailand*

*^3^Biocatalyst and Environmental Biotechnology Research Unit, Department of Biochemistry, Faculty of Science, Chulalongkorn University, Bangkok 10330, Thailand*

*^*^Corresponding author. TR Fax: +66 2 218-5418; Tel: +66 2 218-5426*

*E-mail address:* [*thanyada.r@chula.ac.th*](mailto:thanyada.r@chula.ac.th)


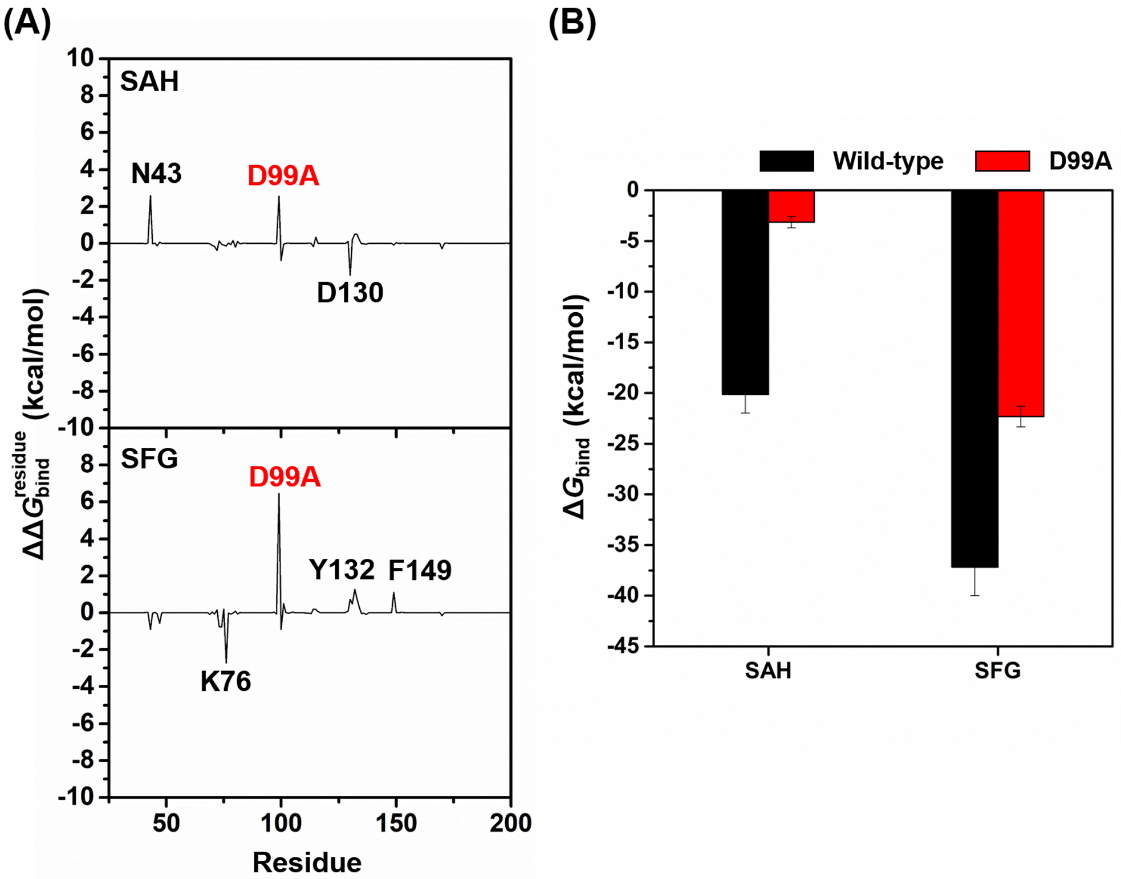


**Fig. S1.** (**A**) $\Delta\Delta G_{\text{bind}}^{\text{residue}}$ of SAH (top) and SFG (bottom) in complex with SARS-CoV-2 nsp16. Note that the $\Delta\Delta G_{\text{bind}}^{\text{residue}}$ was calculated using the following equation: $\Delta\Delta G_{\text{bind}}^{\text{residue}}$= $\Delta G_{\text{bind}}^{\text{residue}}$ (mutant) – $\Delta G_{\text{bind}}^{\text{residue}}$ (wild-type). (**B**) Δ*G*_bind_ of SAH and SFG in complex with wild-type (black) and D99A mutant (red) forms of SARS-CoV-2 nsp16. Data are shown as mean ± standard error of mean. Note that only nsp16/ligand/RNA ternary complex in chain A was considered for free energy calculations.
